# Supplementary material for: Moistube irrigation (MTI) discharge under variable evaporative demand
Source: PLoS One. 2020 Dec 16;15(12):e0236211. doi: 10.1371/journal.pone.0236211 (PMC7743961; doi:10.1371/journal.pone.0236211)
Supplement: S1 Appendix — (DOCX) [file pone.0236211.s001.docx]

**S1 APPENDIX.** Data for the computed $q_{Ave}$ values using Equation 10

| Region | Evaporating power (ET_o_)  mm.d^-1^ | Computed $q_{t}$  l.h^-1^.m^-1^ |
| --- | --- | --- |
| **Humid** | 1 | 0.13 |
|  | 2 | 0.28 |
|  | 3 | 0.43 |
| **Sub-Humid** | 3.5 | 0.51 |
|  | 4 | 0.58 |
|  | 5 | 0.73 |
| **Semi-Arid** | 5.5 | 0.81 |
|  | 6 | 0.88 |
|  | 7 | 1.03 |
| **Arid (**> 7 **mm.d^-1^)** | 7.5 | 1.11 |
|  | 8 | 1.18 |
|  | 9 | 1.33 |
|  | 10 | 1.48 |
