# Supplementary material for: Moistube irrigation (MTI) discharge under variable evaporative demand
Source: PLoS One. 2020 Dec 16;15(12):e0236211. doi: 10.1371/journal.pone.0236211 (PMC7743961; doi:10.1371/journal.pone.0236211)
Supplement: Attachment — Submitted filename: reviwer suggestion.docx [file pone.0236211.s003.docx]

I have read this article titled ‘Moistube™ Irrigation (MTI) Discharge Under Variable Evaporative Demand’ carefully. It’s interesting to research theories of negative pressure irrigation in an artificial evaporative way and the methods it used could be a reference to similar studies in future . However, there existed many problems needing revised completely before taking into consideration for acceptance.

1. Experiments, statistics, and other analyses are not performed to a high technical standard and are not described in sufficient detail as well. For instance, the details of experiment equipments were not clearly described, which was crucial to help readers to understand the paper. Statistics was not performed rigorously and No differences comparisons between treatments.
   2. The article is not written in standard English, and Language is redundance, not concise.
   3. The other suggestions had been labeled on the paper, please check it one by one.

Based upon these considerations, my suggestion is resubmit after major revision.
