# Supplementary material for: Moistube irrigation (MTI) discharge under variable evaporative demand
Source: PLoS One. 2020 Dec 16;15(12):e0236211. doi: 10.1371/journal.pone.0236211 (PMC7743961; doi:10.1371/journal.pone.0236211)
Supplement: Attachment — Submitted filename: Reviewer-Author Log.docx [file pone.0236211.s004.docx]

**PLOSONE Reviewer-Author Comments Log**

**Manuscript**: PONE-D-20-20136

| **Reviewer 1** | | |
| --- | --- | --- |
| **Overall comments:** | | |
| Experiments, statistics, and other analyses are not performed to a high technical standard and are not described in sufficient detail as well. For instance, the details of experiment equipment were not clearly described, which was crucial to help readers to understand the paper. Statistics was not performed rigorously and No differences comparisons between treatments  The article is not written in standard English, and Language is redundancies, not concise. | | |
| **Authors general comment:** | | |
| Reporting on the statistical analysis has been improved (Section 3.2). An improved high-resolution image of the experimental set up has been provided. Explanation on the instrumentation has been reported in detail. A Kruskal-Wallis one-way ANOVA test was employed to compare mean discharges under the various evaporative demand scenarios. The authors have removed redundant statements especially in the conclusion section. | | |
| **Specific comments** | | |
| **Reviewer comments** | **Section in revised document** | **Author response** |
| Abstract should be rewritten with four parts such as Objectives, Methods, Results and conclusions. | Abstract pp. 1 - 2 | The abstract has been reconfigured to follow the suggested format |
| VPD will be greater along with increasing intensity of Ed ,of course. | Abstract pp 2 | Agreed, the statement highlights the basic findings, but we added it to avoid assumptions. |
| These treatments should be clarified by author, it’s not clear. | S 2.2.1 |  |
| This is not standard way of label reference. | S 2.2.1 | The manuscript referencing style has been changed to Vancouver referencing style. |
| The experiment factor should be deemed as u_a_ not E_d_. | S 2.2.1 | The experimental factor has been changed from E_d_ to u_a_. The statement now reads “The experiment was a single factor experiment ($u_{a}$).” |
| Are there duplications to each treatment? | S 2.2.1 pp. 6 Line 132 | Each u_a_ treatment was replicated three times and the resultant q values were the averages of the replications. A supporting statement has been provided. |
| What’s the total time of this experiment? | S 2.2.1 pp. 6 | The three times indicate different run times for each treatment; therefore, the presented times are the overall independent experimental run times. |
| Fig 3 is not clear to readers. A photo maybe better. | S 2.2.2 pp. 7 | A new high-resolution image has been supplied. |
| Where was the experimental equipment made? | S 2.2.2 pp 7 | The authors have included the country of manufacture. |
| What’s difference between resolution and accuracy? | S 2.2.2 pp 7 Line 152 - 153 | The authors have clarified on accuracy and resolution. The edited statement now reads as follows: The relative humidity (RH) was measured using the HCT01-00D sensor (E + E ELEKTRONIC ™, Austria) with a 5 - 95% RH working range, resolution of $\pm$ 2.5% RH, 2% RH accuracy, and a temperature dependency of $\pm$ 0.03% RH/°C, meaning the measured values were $\pm$ 0.02 close to the actual value and a limit of detection of $\pm$ 0.025. |
| These descriptions were not useful to this paper. | S 2.3 pp. 7 – 8 Line 165 - 176 | Duly noted. The authors, however, beg to differ with the reviewer. The statement serves as a preamble leading to the justification of the final selected model. |
| No differences comparison between treatments? | S 2.4 | The comment has been attended to, kindly refer to Section 3.2. |
| What’s the unit? Are there differences significant between them? | S 3.2 pp. 11 Line 246 - 247 | The discharge units have included |
| On the Shapiro-Wilks test: It’s confusing. | S 3.2 pp. 11 Line 246 - 248 | Under Table 3, the $p$ values have been adjusted and presented in a much clearer way i.e., the Shapiro-Wilks $p$ value has been clearly distinguished from the ANOVA $p$ value. |
| This fig should be redrawn for its not suitable ratio between data and axis’s | S 3.2 pp. 13 Line 283 | The figure has been redrawn and it clearly shows the axis and the data points used for the plot. |
| How you calculate t*? the method should be clarified. | S 3.3 pp. 14 Line 311 - 315 | We added Equation 13 to show how we computed the normalised time. |
| Language is redundant, not concise | S 4 pp. 17 Line 367 - 382 | The conclusion has been condensed to remove redundant statements. The conclusion has been separated from the recommendations too. |
| Please edit the references in standard way. | 6 pp. 19 | The manuscript referencing style has been changed to Vancouver referencing style. |
|  |  |  |
| **Reviewer 2** | | |
| **Reviewer comments** | **Section** | **Author response** |
| First of all, the hypothesis is not well defined. The authors are advised to highlight the hypothesis of their work in the broader context so that it can address the general audience. | S 1 pp 5 Line 110 - 112 | The authors improved the hypothesis to be comprehensive. The hypothesis now reads “. The study hypothesized that the presence of an imposed negative pressure or an artificial $E_{d}$ cannot induce MTI discharge i.e., in the absence of a driving pressure and under arid and semi-arid conditions, MTI records zero discharge” |
| The introduction is incomplete and requires a significant overhauling. First, eliminate the equations and address the shortfalls of the previous research works verbally. The equations make it hard to understand how the novel work covers the shortfalls of the previous works. | S 1 pp. 3 - 5 | The authors have converted the equations defining evaporative demand ($E_{d})$into prose. $E_{d}$ has three drivers, which are the hydrological, radiative and the advective limits [5, 7]. For evaporative demand to occur there has to be an adequate water supply to meet the minimum hydrological limit hence, the hydrological limit defines the availability of water to evaporate and transpire from plants and soil surfaces . According to Hobbins and Huntington [5)], the radiative limit is the energy required to facilitate the evaporative process Under field conditions, the radiative limit is influenced by variables such as surface albedo (α), shortwave and longwave radiation. For a buried MTI lateral the soil heat fluxes are also involved. Advection is a critical component for $E_{d}$. The advective limit describes the system boundary’s ability to absorb and bear away moisture.  In addition we added additional text and re arranged some text for lucidity (see track changes). |
| In defining the study area, instead of information about the Laboratory's location, the information on variables used, their characteristics and the variability of these characteristics should be presented. The degree to which these characteristics vary across the study area would have an effect on the results so this should be reported. | S 2.1 | Duly noted, if we understood the comment correctly, we opted for a controlled environment were the measured variables were recorded as such without any variability within the experimental space. The variables in question (RH and temperature) were maintained constant for all experimental treatments. Variability as announced in the comment would be appropriate if it were a field experiment. |
| In the conclusion and recommendation section, the authors have summarised their conclusions in the first paragraph. Instead of deliberating each parameter, they focused on the acceptance/ rejection of the null hypothesis. I recommend the authors to briefly deliberate each selected parameter (or what they call "various evaporative demand scenarios") before coming to the null hypothesis | S 4 pp. 17 Line 372 - 382 | We have rewritten the conclusion by adding a supporting statement that reads:  The steady plateauing discharge curve under low $E_{d}$ (0.16 mm.d^-1^) represented a humid environment where evaporation was occurring on a relatively wet surface. The functional $E_{d}$ - $q$ relationship revealed that at $E_{d}=0$ there is no MTI discharge scenario, therefore, in humid regions there is need to incorporate pumping units to drive irrigation water and effect MTI discharge. Likewise, in arid regions where $E_{d}$ > 7 mm.d^-1^ irrigators can capitalise on the MTI negative pressure discharge capability thus minimising energy costs. |
