# Supplementary material for: Moistube irrigation (MTI) discharge under variable evaporative demand
Source: PLoS One. 2020 Dec 16;15(12):e0236211. doi: 10.1371/journal.pone.0236211 (PMC7743961; doi:10.1371/journal.pone.0236211)
Supplement: Attachment — Submitted filename: Reviewer-Author Comments Log_11_11_2020.docx [file pone.0236211.s005.docx]

**PLOSONE Reviewer-Author Comments Log**

**Manuscript**: PONE-D-20-20136R1

**Note**: The sections herein this document refers to the document with track changes.

| **Overall comments:** | | |
| --- | --- | --- |
| The Moistube irrigation (MTI) system is a very interesting and promising way for the increment of water use efficiency in agriculture, therefore I have crucial concerns about the structure of the manuscript, the description of the experiment and the interpretation of the results. I state some specific objections and some general as well. In the revised manuscript the first equation appears is Equation 5, nevertheless I also read carefully the first submission in order to have a clear aspect. | | |
| **Authors general comment:** | | |
| He authors have taken note and heed of the concerns raised by the reviewer. The Equation numbering has been rectified accordingly and all the queries raised by the reviewer have been attended to the best of our ability. | | |
| **Specific Comments** | |  |
| **Reviewer comments** | **Authors’ response** | **Section in the revised document** |
| 122: dot is missing | The missing dot has been added. | Section 2.2 pp. 5 |
| 127: dot is missing | The missing dot has been added. | Section 2.2 pp. 5 |
| 196: “to be normalised ,t_max_” make it to be “to be normalised, t_max_” | Equation 6 variables have been corrected as per the reviewer’s suggestion. | Page 9, Line 198 - 200 |
| Equation (6): Are not all variables explained (L_u_) | The variable L_u_ has been defined. | Section 2.3.1 pp 8 Line 173 - 174 |
| “The results (Table 2) revealed a positive correlation amongst the variables u_a_,$r$, VPD and E_d_.” I see negative correlation between $r$ and E_d_. | The authors have re-worded the opening statement to:” The results (Table 2) revealed a positive correlation between the variable $u_{a}$, and the other variables $r$ , VPD and $E_{d}$.”.  The authors agree with the reviewer and the negative correlation has been explained. The new paragraph under Table 2 now reads: “The results (Table 2) revealed a positive correlation amongst the variables $u_{a}$, VPD and $E_{d}$. A high air velocity ($u_{a}$) effected a high VPD, which subsequently induced a high $E_{d}$. The observation concurs with Donohue, McVicar [10] whose study attributed high evaporation rates to increased air temperatures. The study established a negative correlation between $r$ and $E_{d}$ which signified that under a low $u_{a}$ evaporation occurred under a relatively and continuously wet surface i.e., wet environment evaporation ($E_{w}$). A high $u_{a}$ increased the drying power of air thus limiting readily available moisture for evaporation thus relatively low $r$ value as compared to the other $r$ values under = 1.2 m.s^-1^ and 2.5 m.s^-1^.” | Section 2.4 pp 10 Line 225 – 227. |
| Line 234-235: This is not understandable. | The statement has been revised and it now reads: “The normality test (Table 3) was carried out on the observed discharge values for each applied evaporative demand scenario ($E_{d}=0.16 {mm.d}^{-1}, 0.31 {mm.d}^{-1} \mathrm{and} 0.36 {mm.d}^{-1}$) The respective sample data revealed high data skewness ($p<{0.05}^{*}$).” | Section 3.2 pp 10, Line 237 - 240 |
| Table 3: “E_d_” make it to be “E_d_” | The mistake has been corrected from Ed to $E_{d}$ | Section 3.2 pp 11, Table 3 |
| The MTI discharge under variable E_d_ was characterized by a power function (R^2^ = 0.62) as shown in Figure 2 over selected time scales. Fig 2 (a) Moistube™ discharge (qt) at 0 ≤ Ed ≤ 7 and (b) comparison between the simulated qt vs the observed qt.” What is this power function? Maybe you refer to Equation 16, so it is confusing for reader while at this point hasn’t seen the equation. I don’t see R^2^=0.62 in Fig. 2. I also don’t see power function but a linear relation between qt and E_d_ | The statement has been rectified to suit the context of the discussion in question. The rectified statement now reads as follows:  “The effect of the negative pressure was determined by establishing a relationship between mass flow rates ($\dot{m}$) against the recorded $E_{d}$. The overall average $q_{t}$ vs $E_{d}$ was modelled as in Equation 12 ($R^{2}=0.55$) as shown in Figure 2 (a) over selected time scales” | Section 3.2 pp 11, Line 248 – 252. |
| I am pretty confused of the procedure you followed. As far as I understand (I’m not sure about that), you calculated the qt with equation: q_t_=0.15E_d_-0.02 according to the regression it seems in Fig. 2. This equation was built by using the average values of measured qt for the respective daily E_d_ values (i.e. 3)? Do you think the number of points you used for the regression modelling are adequate? | The three points used for the model build up were centroid values of an otherwise scattered points for each Ed point, thus the three-point used are centroid values. The statement that reads “for each respective $E_{d}$ sessions, i.e., the $E_{d}$ = 0.16 mm.d^-1^ had 1908 data points, whilst $E_{d}$ = 0.31 mm.d^-1^ had 1608 data points, and $E_{d}$ = 0.36 mm.d^-1^ had 120 data points” explains the number of points required to compute the centroid. | Section 3.2, figure 2 a, Line 258 - 262 |
| Line 292-294: “These observed variations in between = 0.31 mm.d^-1^ and = 0.36 mm.d^-1^ are attributed to high values due to increased drying power of the air in the conduit, hence effecting high discharges.” This is confusing. | The ambiguous statement has been deleted. The revised statement reads “For $E_{d}$ = 0.31 mm.d^-1^ there was a 10% decline in relative discharge ($q_{rel}$) whilst $E_{d}$ = 0.36 mm.d^-1^ recorded a 67% decline in $q_{rel}$ over a $t^{*}$= 1 period. A high $E_{d}$ corresponded to a high $q_{rel}$” | Section 3.2 pp 13, Line 300 – 303. |
| In Figure 3 points of different kinds should be corrected. | The graphical markers have been corrected i.e.; the individual plot markers match the plot markers on the combined $E_{d}$ graphs. | Section 3.3 pp 16, Line 377 |
| It is not clarified well the graph you refer to (a, b, c or d). There are divergencies between the values that are referred and Figure 3.  Concerning the “Results and Discussion” section, there are ambiguities which confuse the reader. I consider that the experiment was extensive, but I actually didn’t understand very well the interpretation of the results | For the avoidance of ambiguity and confusion, the authors have re-worked some sections of the discussion. For example, **Section 3.3 pp 15 Line 334 – 349** constitutes an incisive and straight forward result interpretations. The material has been revised to communicate with graphical presentations. Also, the authors have corrected the information presented in **Section 3.3 pp 16, Line 362 – 366** to match the graphical data (**Figure 3 a**).  The discussion has been compartmentalised into specific discussion items/points to avoid an ambiguity. | Section 3.3 pp 15 Line 334 – 349  Section 3.3 pp 16, Line 362 – 366 |
| How the applicability and performance of the particular experiment is connected to the maintenance of soil water at 80-90% of the field capacity levels? At this point field capacity is referred for the first time. | The assertion has been rectified and it now reads as follows:” MTI is conceptually a continuous irrigation technique which potentially maintains the soil at field capacity (FC) level thus, it is capable of maintaining soil moisture levels at FC. This is instrumental in availing adequate crop water without having to fully saturate the soil [[30](https://stuukznac-my.sharepoint.com/personal/215081795_stu_ukzn_ac_za/Documents/PhD/PhD%20Chapters/Chapter%204_Evaporative%20Demand/PLOS%20ONE/Revision%202.0/Dirwai_et_al_2020_MTI%20%20Evap_Demand_05_11_2020.docx#_ENREF_30)]” | Section 4 pp 17, Line 390 - 392 |
